# Information-Theoretic Intrinsic Motivation for Reinforcement Learning in Combinatorial Routing

**DOI:** 10.3390/e28020140

**Published:** 2026-01-27

**Authors:** Ruozhang Xi, Yao Ni, Wangyu Wu

**Affiliations:** 1 Krieger School of Arts and Sciences, Johns Hopkins University, Washington, DC 20001, USA; rxi4@jh.edu; 2 School of Integrated Circuit Engineering, Guangdong University of Technology, Guangzhou 510006, China; 3 School of Computer Science, University of Liverpool, Liverpool L69 3DR, UK

**Keywords:** intrinsically-motivated reinforcement learning, information bottleneck, curiosity-driven exploration, combinatorial routing problems

## Abstract

Intrinsic motivation provides a principled mechanism for driving exploration in reinforcement learning when external rewards are sparse or delayed. A central challenge, however, lies in defining meaningful novelty signals in high-dimensional and combinatorial state spaces, where observation-level density estimation and prediction-error heuristics often become unreliable. In this work, we propose an information-theoretic framework for intrinsically motivated reinforcement learning grounded in the Information Bottleneck principle. Our approach learns compact latent state representations by explicitly balancing the compression of observations and the preservation of predictive information about future state transitions. Within this bottlenecked latent space, intrinsic rewards are defined through information-theoretic quantities that characterize the novelty of state–action transitions in terms of mutual information, rather than raw observation dissimilarity. To enable scalable estimation in continuous and high-dimensional settings, we employ neural mutual information estimators that avoid explicit density modeling and contrastive objectives based on the construction of positive–negative pairs. We evaluate the proposed method on two representative combinatorial routing problems, the Travelling Salesman Problem and the Split Delivery Vehicle Routing Problem, formulated as Markov decision processes with sparse terminal rewards. These problems serve as controlled testbeds for studying exploration and representation learning under long-horizon decision making. Experimental results demonstrate that the proposed information bottleneck-driven intrinsic motivation improves exploration efficiency, training stability, and solution quality compared to standard reinforcement learning baselines.

## 1. Introduction

Intrinsic motivation has been widely studied as a mechanism for guiding exploration in reinforcement learning, particularly in settings where task-specific rewards are sparse or delayed. In long-horizon decision-making problems, agents often receive meaningful feedback only after executing a sequence of actions, which makes exploration driven solely by extrinsic rewards highly inefficient. In this context, intrinsic motivation refers to the design of auxiliary reward signals that provide dense, task-agnostic feedback during intermediate decision steps, encouraging the agent to explore informative state–action transitions even in the absence of immediate external rewards. Rather than random exploration, such intrinsic signals aim to guide exploration toward experiences that are expected to improve the agent’s internal understanding of the environment.

This challenge is especially pronounced in reinforcement learning for combinatorial optimization, where the state and action spaces grow combinatorially and rewards are typically available only upon completion of a full solution. Although reinforcement learning has been successfully applied to problems such as the Travelling Salesman Problem and vehicle routing variants, effective exploration in these settings remains nontrivial. Common intrinsic reward designs based on raw observation novelty, prediction error, or state visitation counts often become unreliable in high-dimensional and combinatorial environments, as they are sensitive to irrelevant variability and suffer from the curse of dimensionality [1,2]. In this work, we do not aim to introduce a new reinforcement learning formulation for routing problems. Instead, we focus on how information-theoretic intrinsic motivation can be used to improve exploration efficiency in long-horizon combinatorial decision processes by defining intrinsic rewards that are grounded in task-relevant representations rather than raw observations.

From an information-theoretic perspective, effective exploration requires representations that retain task-relevant structure while discarding nuisance variability. This naturally motivates the use of the Information Bottleneck (IB) principle as a normative framework for representation learning in reinforcement learning [3]. The IB principle formalizes a trade-off between compression and prediction: a representation should be as compact as possible while preserving information that is relevant for predicting future states and outcomes. In the context of exploration, such representations provide a meaningful latent space in which novelty and uncertainty can be quantified in a principled manner.

Concretely, let $O_{t}$ denote the observed environment state at time *t*, $A_{t}$ the chosen action, and $S_{t}$ a learned latent representation of the observation. The IB objective seeks to learn a compact representation by minimizing the mutual information between the observation and its latent encoding, $I(O_{t};S_{t})$, while preserving information that is predictive of future dynamics by maximizing the mutual information between the current latent–action pair and the next latent state, $I(S_{t+1};\left\{S_{t},A_{t}\right\})$. This trade-off can be expressed as

(1) $$L=I\left(O_{t};S_{t}\right)-\beta I\left(S_{t+1};\left\{S_{t},A_{t}\right\}\right),$$

where the first term encourages abstraction and compression of high-dimensional observations, and the second term ensures that the learned representation retains task-relevant information for predicting environment transitions. By grounding representation learning in this objective, intrinsic motivation can be defined in terms of information gain in a structured latent space, rather than superficial novelty in the observation space.

In Equation (1), the trade-off parameter β controls the balance between compression of the state representation and preservation of task-relevant information. From an information-theoretic perspective, larger values of β enforce stronger compression by penalizing mutual information between the input state and the latent representation, leading to more abstract and invariant latent codes. Conversely, smaller values of β retain finer-grained details of the state at the cost of increased redundancy.

In the context of combinatorial routing problems, excessive compression may remove information necessary for distinguishing structurally different partial solutions, while insufficient compression may lead to latent representations that are sensitive to irrelevant combinatorial variability. We therefore adopt moderate values of β that encourage compact yet predictive representations, which empirically results in latent spaces that are both stable and informative for downstream exploration.

Grounded in the IB principle, intrinsic motivation can thus be interpreted as a drive to acquire informative state-action transitions under a compact and task-relevant representation. Rather than assigning curiosity based on raw state novelty or prediction error, an information-theoretic formulation allows intrinsic rewards to be defined in terms of mutual information. Intuitively, a transition is intrinsically valuable if it reveals new information about the environment dynamics that is not already captured by the agent’s current representation. This view is consistent with earlier information-theoretic approaches to curiosity and exploration, which characterize intrinsic motivation as the maximization of predictive information or the reduction of uncertainty about future states [4,5,6].

A critical implication of this formulation is that novelty should be evaluated in an appropriate representation space rather than directly in the observation space. In high-dimensional environments, raw observations often contain substantial nuisance variability that is irrelevant to decision making, such as perceptual redundancy, background noise, or combinatorial symmetries. As a result, density-based or count-based novelty measures defined on observations can be highly misleading and fail to reflect meaningful uncertainty [7,8]. By contrast, representations learned under the Information Bottleneck objective explicitly filter out irrelevant variability while preserving information necessary for predicting future states. Evaluating intrinsic rewards in such a latent space yields a notion of novelty that is both scalable and semantically meaningful, particularly in long-horizon decision problems where early exploration decisions can have lasting consequences.

While reinforcement learning has achieved strong empirical performance in combinatorial routing through attention-based and graph-based architectures [9,10], most existing methods rely exclusively on sparse task-specific rewards. This makes exploration increasingly challenging as problem scale and decision horizon grow. Although intrinsic motivation has been extensively studied in reinforcement learning, its application to combinatorial optimization remains underexplored, particularly from an information-theoretic perspective that explicitly accounts for representation compression and predictive structure.

In this work, we build on these insights and propose an information-theoretic framework for intrinsically motivated reinforcement learning grounded in the IB principle. Intrinsic rewards are defined in terms of mutual information computed in a bottlenecked latent space, enabling principled and scalable exploration without relying on observation-level novelty. We evaluate the proposed approach on two representative combinatorial routing problems: the Travelling Salesman Problem and the Split Delivery Vehicle Routing Problem, both of which are formulated as Markov decision processes with sparse terminal rewards. These problems serve as controlled testbeds for studying exploration and representation learning under long-horizon decision making.

To summarize, our main contributions are summarized as follows:

- We propose an information-theoretic framework for intrinsically motivated reinforcement learning grounded in the IB principle, providing a unified view of representation learning and exploration.
- We formulate intrinsic rewards in terms of pointwise mutual information within a bottlenecked latent space, enabling novelty to be quantified as information gain rather than observation-level dissimilarity.
- We develop a practical learning framework that integrates representation learning and intrinsic motivation without relying on explicit density modeling or contrastive objectives.
- We empirically demonstrate the effectiveness of the proposed approach on combinatorial routing problems with sparse rewards, showing improved exploration efficiency, training stability, and solution quality.

## 2. Related Work

Curiosity-driven reinforcement learning typically relies on two tightly coupled components: the design of intrinsic reward signals that quantify novelty or informativeness, and the learning of state representations that suppress irrelevant variability while preserving task-relevant structure. These two aspects jointly determine whether intrinsic motivation provides meaningful guidance for exploration. As prior work has emphasized different facets of this problem, we review related studies by first focusing on the design of intrinsic rewards, followed by representation learning for curiosity-driven exploration.

### 2.1. Intrinsic Reward Design

A large body of prior work defines intrinsic rewards based on prediction-based signals, where curiosity is associated with the agent’s inability to accurately predict future outcomes. Methods such as Intrinsic Curiosity Modules (ICM) [7] and Random Network Distillation (RND) [8] operationalize this idea by using prediction error as the intrinsic reward, measured as the discrepancy between predicted and observed targets. While ICM predicts the next environment state, RND compares the output of a learned predictor against that of a fixed random network. These approaches encourage exploration toward regions where the agent’s predictive model is inaccurate.

Beyond pointwise prediction error, several works quantify curiosity through model disagreement or uncertainty. Disagreement-based exploration [11] assigns intrinsic rewards according to the variance among predictions from an ensemble of dynamics models, reflecting epistemic uncertainty. Related uncertainty-aware techniques, including Monte Carlo dropout [12] and deep ensembles [13], similarly estimate uncertainty by measuring variability in model outputs. Such methods promote exploration of state-action regions where the agent’s predictions are uncertain or inconsistent.

Another prominent line of work is based on learning progress, which is conceptually aligned with psychological theories of curiosity. Rather than rewarding prediction error itself, these methods focus on the temporal evolution of learning signals, encouraging exploration in regions where the agent’s predictive ability improves most rapidly. For example, progress-based rewards have been defined using changes in transition probabilities [14] or derivatives of prediction error over time [15]. VIME [16] formalizes this idea from a Bayesian perspective by measuring the Kullback–Leibler divergence between posterior and prior distributions of a learned dynamics model, interpreting learning progress as information gain about model parameters. Similarly, diversity-driven exploration [17] encourages exploration by rewarding substantial changes in the agent’s policy distribution over time.

From a broader information-theoretic viewpoint, many of these intrinsic reward formulations can be interpreted as approximations of uncertainty reduction or information gain. Early work such as DIDO [18] explicitly employed Shannon entropy to guide exploration toward state–action pairs with high epistemic uncertainty. More recent approaches likewise aim to identify experiences that are maximally informative for improving the agent’s internal model, even when this connection to information theory is implicit. These observations motivate the search for intrinsic reward formulations that directly quantify informativeness in a principled, information-theoretic manner.

### 2.2. Information-Theoretic Representation Learning in Reinforcement Learning

A central challenge in curiosity-driven reinforcement learning is to learn state representations that are simultaneously compact, predictive, and relevant for control. Information-theoretic objectives provide a principled framework for addressing this challenge by explicitly characterizing what information a representation should retain or discard. In this subsection, we review prior work that employs information-theoretic criteria for representation learning in reinforcement learning, with a focus on their implications for exploration.

Several studies have investigated the use of mutual information to extract representations that capture environment dynamics while suppressing task-irrelevant variability [19,20]. Related approaches apply the IB principle to reinforcement learning, aiming to balance compression of observations with preservation of information relevant for predicting future states and rewards [3,21,22]. These works highlight the importance of learning latent representations that are informative for decision making without being tied to raw observation statistics.

A complementary line of research emphasizes representation sufficiency for control. From this perspective, a representation is desirable if it preserves value-relevant structure while being invariant to nuisance factors. This intuition is formalized through bisimulation-based metrics, which consider states equivalent when they induce similar rewards and transition dynamics. Methods such as DeepMDP and subsequent invariant representation learning approaches leverage this idea to learn latent spaces that are robust to irrelevant visual variations while remaining suitable for planning and control [23,24].

Predictive representation learning has also been widely studied in the context of model-based reinforcement learning. World-model approaches learn latent state-space dynamics optimized to predict future observations and rewards, enabling planning directly in the learned representation space [25,26,27]. While such objectives implicitly encourage strong dependence between successive latent states conditioned on actions, they typically do not explicitly control the trade-off between compression and prediction, and may therefore retain redundant or nuisance information that is not essential for efficient exploration.

Mutual information maximization has further been explored in unsupervised and self-supervised representation learning, including contrastive formulations. Methods such as Contrastive Predictive Coding and Deep InfoMax estimate tractable lower bounds on mutual information by distinguishing samples from the joint distribution and the product of marginals [28,29]. In reinforcement learning, these ideas have been adapted to encourage representations that emphasize decision-relevant features or temporally predictive structure. Despite strong empirical performance, many such approaches rely on contrastive objectives whose connection to intrinsic motivation remains indirect.

More closely related to curiosity-driven exploration, several works explicitly link intrinsic motivation to information gain in a learned latent space, aiming to construct representations that are predictive of controllable dynamics and to define curiosity signals based on changes in the agent’s internal model [19,20]. While differing in implementation, they share the intuition that exploration should be guided by how much an experience improves the agent’s knowledge about the environment, rather than by surface-level novelty in the observation space.

In contrast to prior work, our approach adopts the IB principle as a unifying framework for representation learning and intrinsic motivation. We explicitly enforce a trade-off between compression and predictiveness in the learned latent space and define intrinsic rewards directly in terms of mutual information associated with state-action transitions. By grounding both representation learning and exploration in the same information-theoretic objective, our method provides a principled alternative to heuristic or purely contrastive formulations of curiosity.

### 2.3. Reinforcement Learning for Solving Combinatorial Routing Problems

Combinatorial routing problems such as the Travelling Salesman Problem (TSP) and Vehicle Routing Problems (VRPs) are classical NP-hard optimization problems with broad relevance in logistics and transportation. Decades of operations research have developed strong exact solvers and heuristics, including branch-and-bound/cutting-plane methods and local search strategies such as Lin–Kernighan–Helsgaun (LKH), which remain highly competitive for many practical instances [30,31]. These approaches, however, are typically hand-engineered and may require substantial effort to adapt across variants, constraints, and instance distributions.

Recently, learning-to-optimize paradigms have emerged as an alternative that aims to amortize the cost of solving by learning policies or constructive procedures that directly output feasible solutions. Early neural approaches introduced pointer-network style architectures to generate tours for routing problems [32,33]. Subsequent work demonstrated that reinforcement learning can train such models end-to-end without supervised labels, enabling generalization across instance sizes and distributions while optimizing solution quality directly [33]. A representative line of work adopts attention-based or graph-based encoders and trains constructive policies for TSP/VRP via policy gradient or actor-critic methods [9,10].

For VRP variants with capacity or demand constraints, reinforcement learning has been applied to sequentially construct routes by choosing the next customer (or returning to the depot) while maintaining feasibility through state augmentation and masking. For example, Nazari et al. [9] proposed an RL framework for VRP that incorporates dynamic demand and capacity information into the policy input, while Kool et al. [10] developed an attention-based model that achieves strong performance on multiple routing problems and supports efficient batched decoding. Beyond purely constructive decoding, hybrid methods combine learned policies with classical improvement operators (e.g., 2-opt) or local search to refine candidate solutions, often yielding better solution quality while retaining generalization [34].

Compared to IBE [35], which incorporates information bottlenecks at the state and policy levels as regularization terms within the policy optimization objective, our approach differs in both motivation and mechanism. Specifically, IBE influences exploration implicitly through policy-level regularization, without constructing an explicit intrinsic reward signal that augments the environment reward. In contrast, VIB-IG defines information gain in the latent representation space as a standalone intrinsic reward, which is directly added to the return and shapes the agent’s exploration behavior. This distinction allows VIB-IG to decouple representation learning from policy optimization and to explicitly target exploration under sparse and delayed rewards.

Learning-based routing methods can be naturally formulated as MDPs, where the state encodes the current partial solution and remaining constraints (e.g., unvisited nodes, residual demands, and remaining capacity), the action corresponds to the next routing decision (e.g., selecting the next node to visit or determining the amount of demand to serve), and the episode terminates once a feasible complete solution is constructed. Such formulations typically induce sparse and delayed reward signals, as the optimization objective (e.g., total route length) can only be evaluated upon completion of the solution. This makes routing problems a controlled yet challenging testbed for studying exploration and representation learning in reinforcement learning, particularly in environments with high-dimensional or combinatorial state spaces.

## 3. Method

### 3.1. Problem Formulation and Overview

We consider combinatorial routing problems, such as the Travelling Salesman Problem (TSP) and the Split Delivery Vehicle Routing Problem (SDVRP), formulated as finite-horizon MDPs. In this setting, an agent sequentially interacts with an environment by observing the current state and selecting actions in order to construct a feasible routing solution.

At each discrete time step *t*, the agent observes the current environment state $O_{t}$, which encodes a partial routing solution, including information such as the set of already visited nodes, remaining customer demands, vehicle capacity, and the current location of the vehicle. Based on this observation, the agent selects an action $A_{t}$ according to a stochastic policy $\pi (A_{t}|O_{t})$. In routing problems, actions typically correspond to discrete routing decisions, such as selecting the next customer to visit, assigning a customer to a specific vehicle, or deciding whether to return to the depot. Executing an action extends the partial solution and leads to the next observation $O_{t+1}$.

The objective of the agent is to learn a policy that maximizes the expected cumulative reward over an episode. An episode terminates once a feasible complete routing solution is constructed. At this point, a task-specific extrinsic reward is provided based on the quality of the final solution, for example the negative total route length or total transportation cost. Importantly, during most intermediate steps of the decision process, no informative task-level feedback is available. As a result, routing problems naturally induce sparse and delayed reward signals, since meaningful evaluation of solution quality is only possible after a long sequence of routing decisions has been completed.

This delayed-feedback structure poses a significant challenge for standard reinforcement learning methods. The agent must explore a combinatorially large decision space and commit to long action sequences before receiving any indication of whether its choices were beneficial. Consequently, exploration driven solely by extrinsic rewards is highly inefficient and often leads to premature convergence to suboptimal routing strategies.

To address this challenge, we introduce an intrinsically motivated reinforcement learning framework that augments the sparse extrinsic reward with an intrinsic signal designed to encourage informative exploration. The intrinsic reward provides dense feedback during intermediate decision steps, guiding the agent toward actions that improve its understanding of the environment dynamics and the structure of high-quality routing solutions. In contrast to standard Proximal Policy Optimization (PPO) [36] trained solely with terminal rewards, the intrinsic signal provides step-wise guidance that reduces the effective exploration horizon.

**Figure 1:**
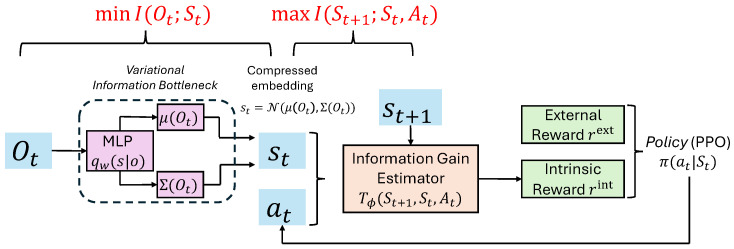
Overview of the proposed intrinsically motivated reinforcement learning framework. Observations $O_{t}$ are encoded into a bottlenecked latent state $S_{t}$ via a variational Information Bottleneck objective that simultaneously minimizes $I(O_{t};S_{t})$ to encourage compression and maximizes $I(S_{t+1};S_{t},A_{t})$ to preserve information relevant for predicting future dynamics. Based on the learned latent representation, a neural information gain estimator computes the pointwise information gain between the current state-action pair $\left(S_{t},A_{t}\right)$ and the next latent state $S_{t+1}$, yielding an intrinsic reward signal. The intrinsic reward complements sparse task-specific extrinsic rewards and is integrated into a PPO-based policy optimization loop.

Our approach consists of three key components: (i) learning a compact and task-relevant latent state representation via the Information Bottleneck principle, (ii) defining intrinsic rewards in the learned latent space based on information gain, and (iii) optimizing the policy using a standard policy-gradient algorithm. An overview of the proposed framework is illustrated in Figure 1.

### 3.2. Information Bottleneck for State Representation

A core component of our framework is the learning of a compact latent state representation that preserves information relevant for decision making while discarding nuisance variability in the raw observations. To this end, we adopt the IB principle to learn a bottlenecked latent representation.

Let $O_{t}$ denote the observation at time *t*, and let $S_{t}$ be the corresponding latent state obtained via an encoder network $q_{\omega }\left(S_{t}|O_{t}\right)$. Given the action $A_{t}$ taken at time *t*, the environment transitions to the next latent state $S_{t+1}$. The objective of representation learning is to compress the observation $O_{t}$ into $S_{t}$ while retaining sufficient information to predict the future dynamics.

Formally, we optimize the following Information Bottleneck objective:

(2) $$L_{\mathrm{IB}}=I\left(O_{t};S_{t}\right)-\beta I\left(S_{t+1};S_{t},A_{t}\right),$$

where the first term encourages compression of the observation into a low-dimensional latent space, and the second term enforces that the latent representation preserves information relevant for predicting future state transitions.

In principle, the compression term $I(O_{t};S_{t})$ measures the mutual information between observations and latent states and can be written as

(3) $$I\left(O_{t};S_{t}\right)=E_{p(O_{t})}\left[\mathrm{KL}\;\left(p\left(O_{t},S_{t}\right)\;∥\;p\left(O_{t}\right)p\left(S_{t}\right)\right)\right].$$

Directly optimizing this quantity is intractable, as the joint distribution $p(O_{t},S_{t})$ is unknown. Following the variational Information Bottleneck (VIB) formulation [21], we instead minimize an upper bound on $I(O_{t};S_{t})$ by introducing a variational encoder $q_{\omega }\left(S_{t}|O_{t}\right)$ and a prior $p(S_{t})$:

(4) $$I\left(O_{t};S_{t}\right)\;\leq \;E_{p(O_{t})}\;\left[\mathrm{KL}\;\left(q_{\omega }\left(S_{t}|O_{t}\right)\;∥\;p\left(S_{t}\right)\right)\right].$$

In practice, we model $q_{\omega }\left(S_{t}|O_{t}\right)$ as a Gaussian distribution with mean $\mu_{\omega }\left(O_{t}\right)$ and diagonal covariance $\sigma^{2}I$, and adopt a standard normal prior $p\left(S_{t}\right)=N\left(0,I\right)$. Under this assumption, the compression term admits a closed-form expression:  

(5) $$L_{\mathrm{comp}}=\frac{1}{2}\sum_{j=1}^{d}\left(\mu_{\omega ,j}{\left(O_{t}\right)}^{2}+\sigma^{2}-1-\log\sigma^{2}\right),$$

where *d* denotes the dimensionality of the latent state.

The predictive term $I(S_{t+1};S_{t},A_{t})$ is estimated using a neural mutual information estimator, as described in Section 3.3.

Importantly, the IB objective is used solely for representation learning and does not directly define the reinforcement learning reward. Optimizing this objective yields a latent space whose geometry reflects task-relevant environment dynamics, making distances and information-theoretic quantities semantically meaningful. The IB therefore shapes the latent space in which information gain is evaluated, indirectly determining the intrinsic reward.

### 3.3. Mutual Information Estimation

Both the IB objective and the intrinsic reward defined in the subsequent section involve mutual information between continuous random variables. In particular, our framework requires estimating the mutual information between the next latent state and the current state-action pair, $I(S_{t+1};S_{t},A_{t})$. Among various mutual information estimators, we adopt the Mutual Information Neural Estimator (MINE) [37], which is based on variational lower bounds, due to its suitability for online reinforcement learning and intrinsic reward computation.

MINE builds upon the Donsker–Varadhan (DV) representation of the Kullback–Leibler divergence and introduces a parametric function $T_{\phi }\left(\cdot \right)$ to approximate the log-density ratio between the joint distribution and the product of marginals. Specifically, for random variables *X* and *Y*, mutual information can be expressed as

(6) $$I\left(X;Y\right)=E_{p(x,y)}\;\left[T_{\phi }\left(x,y\right)\right]-\log E_{p(x)p(y)}\;\left[\exp(T_{\phi }\left(x,y\right))\right].$$

In our setting, we instantiate $X=(S_{t},A_{t})$ and $Y=S_{t+1}$. The MI critic $T_{\phi }\left(S_{t+1},S_{t},A_{t}\right)$ is trained using mini-batches of trajectory samples. Joint samples are constructed from actual transitions $\left(S_{t},A_{t},S_{t+1}\right)$ observed in the environment, while samples from the product of marginals are obtained by pairing $\left(S_{t},A_{t}\right)$ with randomly permuted next latent states ${\tilde{S}}_{t+1}$ within the same mini-batch, thereby breaking temporal dependence.

Optimizing the above objective encourages $T_{\phi }$ to approximate the pointwise log-density ratio

(7) $$T_{\phi }\left(S_{t+1},S_{t},A_{t}\right)\;\approx \;\log\frac{p(S_{t+1}|S_{t},A_{t})}{p(S_{t+1})},$$

which corresponds to the information gain of an individual transition. This property enables the MI critic to be evaluated at the level of single state–action transitions and subsequently used to define intrinsic rewards.

Importantly, MINE serves as a numerical approximation tool for mutual information and information gain. It does not alter the underlying information-theoretic definitions and can be trained online using samples collected during policy rollouts.

**Remark** **1.** *Several alternative approaches exist for estimating mutual information. Classical non-parametric estimators, such as k-nearest neighbors (KNN) [38] or kernel density estimation (KDE), rely on local density estimation and require careful tuning of hyperparameters (e.g., the number of neighbors or kernel bandwidth). In high-dimensional latent spaces, which naturally arise in reinforcement learning, these estimators often suffer from severe bias and instability.*

*Recent contrastive estimators, including InfoNCE and Jensen–Shannon-based objectives [28,29], estimate mutual information by discriminating between positive and negative sample pairs. While effective in representation learning, such methods require the explicit construction of negative samples and large batch sizes to achieve tight bounds, which complicates their integration into online reinforcement learning and intrinsic reward computation.*

*In contrast, MINE directly learns a parametric approximation of the log-density ratio via a variational bound on mutual information [37]. This formulation avoids explicit density modeling and does not require hand-crafted kernel bandwidths or explicit positive-negative pairing. Crucially, the learned MI critic provides a pointwise score that can be evaluated at the level of individual transitions, making it well-suited for defining information-gain-based intrinsic rewards, as will be discussed in Section 3.4.*

**Remark** **2.** *Several modern contrastive objectives, such as InfoNCE and Contrastive Predictive Coding (CPC) [28], have been widely used for representation learning by maximizing a lower bound on mutual information via negative sampling. While effective in stationary and large-batch settings, these estimators rely critically on the construction of informative negative samples and the assumption of relatively stable data distributions.*

*In reinforcement learning for combinatorial optimization, however, the state distribution is inherently non-stationary due to continual policy updates, and the notion of meaningful negatives becomes ambiguous for intermediate decision states. As a result, contrastive estimators may exhibit instability or introduce bias during training. In contrast, MINE directly estimates mutual information by contrasting samples from the joint distribution with samples from the product of marginals, without requiring explicit negative sampling. This property makes MINE more robust and better suited for estimating information gain in evolving latent spaces induced by reinforcement learning.*

In our experiments, we found that MINE remained numerically stable due to the relatively low-dimensional latent space enforced by the Information Bottleneck and the use of running normalization for intrinsic rewards.

### 3.4. Intrinsic Reward Definition

Intrinsic motivation in our framework is defined in the learned bottlenecked latent space. Let $S_{t}$ and $S_{t+1}$ denote the latent states at consecutive time steps, and let $A_{t}$ be the action taken at time *t*. We define the intrinsic reward as the information gain provided by an individual state-action transition:

(8) $$r_{t}^{\mathrm{int}}=\log\frac{p(S_{t+1}|S_{t},A_{t})}{p(S_{t+1})}.$$

This quantity measures how informative a single transition is about the environment dynamics under the learned representation, and its expectation over the trajectory distribution corresponds to the mutual information $I(S_{t+1};S_{t},A_{t})$. In practice, this information gain is approximated using the learned neural mutual information critic $T_{\phi }\left(S_{t+1},S_{t},A_{t}\right)$ introduced in Section 3.3, which provides a pointwise estimate of the log-density ratio.

By computing intrinsic rewards in the bottlenecked latent space, we ensure that novelty and informativeness are evaluated with respect to task-relevant structure rather than raw observation noise. The intrinsic reward is used exclusively to encourage exploration and does not replace the task-specific extrinsic reward.

### 3.5. Learning Algorithm

Policy optimization in our framework is performed using Proximal Policy Optimization (PPO) [36], a widely used on-policy actor-critic algorithm. PPO improves training stability by constraining policy updates through a clipped surrogate objective, while retaining the simplicity of first-order optimization.

In our framework, PPO serves as the policy optimization backbone and is used without architectural or objective modifications. The policy is trained to maximize the expected cumulative reward, where the reward signal combines task-specific extrinsic rewards and intrinsic rewards derived from information gain.

Specifically, at each time step *t* the agent receives a combined reward

(9) $$r_{t}=r_{t}^{\mathrm{ext}}+\lambda \;r_{t}^{\mathrm{int}},$$

where $r_{t}^{\mathrm{ext}}$ denotes the task-specific extrinsic reward and $r_{t}^{\mathrm{int}}$ is the intrinsic reward defined in Section 3.4. The coefficient λ controls the relative strength of intrinsic motivation and is annealed during training to gradually shift the learning focus from exploration to task optimization. Following common practice in intrinsically motivated reinforcement learning, the intrinsic reward weight λ is annealed during training to gradually shift the learning focus from exploration to task optimization. In our implementation, λ is linearly annealed from an initial value $\lambda_{0}=0.5$ to zero over the course of training.

PPO updates the policy parameters by maximizing the clipped surrogate objective

(10) $$L_{\mathrm{PPO}}=E_{t}\left[\min\;\left(r_{t}\left(\theta \right)\;{\hat{A}}_{t},\;\mathrm{clip}\;\left(r_{t}\left(\theta \right),1-\epsilon ,1+\epsilon \right){\hat{A}}_{t}\right)\right],$$

where $r_{t}\left(\theta \right)=\pi_{\theta }\left(A_{t}|O_{t}\right)/\pi_{\theta_{\mathrm{old}}}\left(A_{t}|O_{t}\right)$ denotes the probability ratio between the updated and previous policies, ${\hat{A}}_{t}$ is the advantage estimate, and ϵ controls the size of the trust region.

The intrinsic reward is normalized using running statistics to stabilize policy optimization. Advantages are computed using Generalized Advantage Estimation with the combined reward signal, and the policy and value networks are updated using the standard PPO clipped objective.

Based on the above formulation, we refer to the proposed framework as VIB-IG, short for Variational Information Bottleneck with Information Gain, which integrates bottlenecked representation learning with information-gain–driven intrinsic motivation. Algorithm 1 summarizes the overall training procedure of the proposed VIB-IG framework. Each training epoch alternates between three tightly coupled components: (i) variational Information Bottleneck representation learning, which learns a compact and predictive latent state $S_{t}$ by minimizing $L_{\mathrm{IB}}$; (ii) variational mutual information estimation, which trains the MI critic $T_{\phi }$ to approximate the pointwise information gain of state-action transitions; and (iii) policy optimization via PPO, where the intrinsic reward computed from $T_{\phi }$ is combined with sparse extrinsic rewards to guide exploration. The intrinsic reward weight λ is annealed linearly during training to gradually shift the learning focus from exploration to task optimization. Specifically, at training epoch *t*, λ is updated as

(11) $$\lambda_{t}=\lambda_{0}\cdot \max\left(0,\;1-\frac{t}{T}\right),$$

where $\lambda_{0}$ denotes the initial intrinsic reward weight and *T* is the total number of training epochs. Unless otherwise stated, we set $\lambda_{0}=0.5$ in all experiments. We also provide an ablation study in Section 4.6.3.
**Algorithm 1:** VIB-Driven Information-Gain PPO
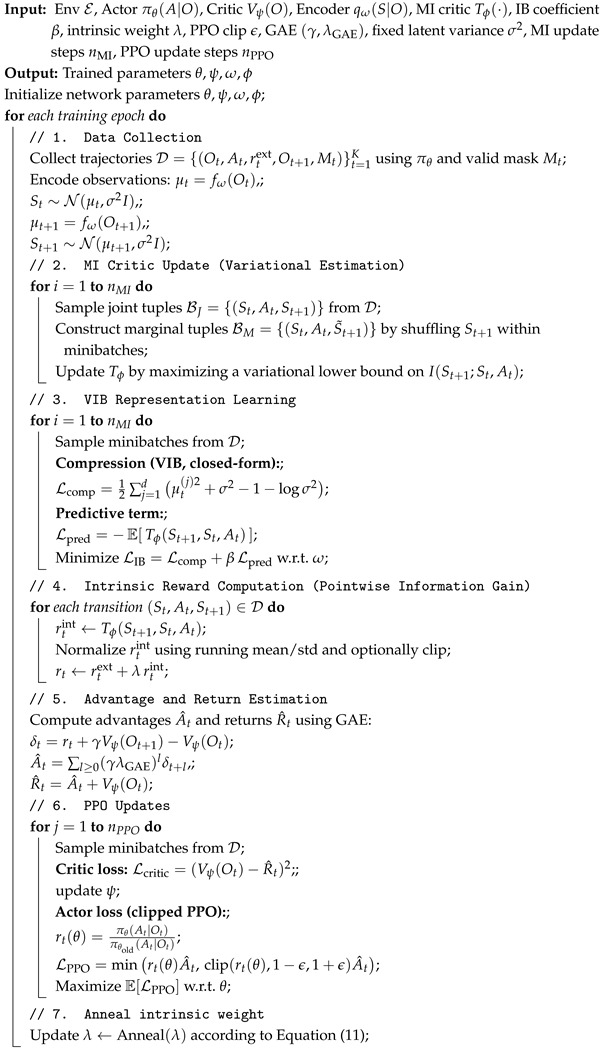


## 4. Experiments

In this section, we empirically evaluate the proposed VIB-IG framework on combinatorial routing problems with sparse terminal rewards. We compare VIB-IG against standard reinforcement learning baselines built upon Proximal Policy Optimization (PPO), as well as representative intrinsic-motivation variants, to assess the effectiveness of information-theoretic exploration.

We begin by describing the experimental environments and evaluation protocol, followed by quantitative comparisons on routing benchmarks of varying scales. We then present analyses of learning dynamics and ablation studies to isolate the contributions of the Information Bottleneck and information-gain-based intrinsic reward components.

Through these experiments, we aim to address the following research questions:

1. How does VIB-IG perform on combinatorial routing tasks compared with standard PPO-based baselines under sparse and delayed rewards?
2. What is the contribution of each component in VIB-IG, particularly the Information Bottleneck representation and the information-gain intrinsic reward, to overall performance and training stability?
3. How does the intrinsic reward influence exploration behavior and policy learning dynamics over the course of training?

### 4.1. Problem Statement

**Figure 2:**
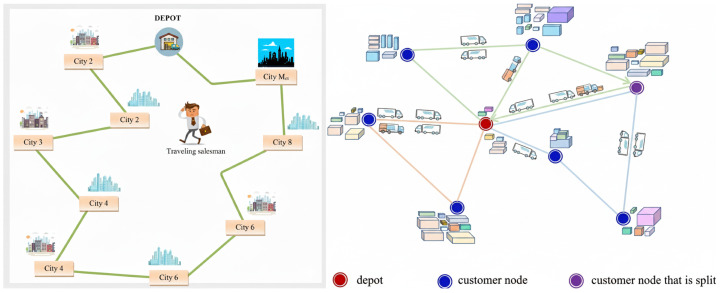
Visual comparison of the two routing environments considered in this work. Left: Travelling Salesman Problem (TSP), where each city is visited exactly once. Right: Split Delivery Vehicle Routing Problem (SDVRP), where customer demands may be fulfilled over multiple visits.

Following prior work in reinforcement learning for combinatorial optimization (RL4CO) [39], we formulate routing problems as episodic MDPs. In this work, we focus on two representative routing tasks: the Travelling Salesman Problem (TSP) and the Split Delivery Vehicle Routing Problem (SDVRP). Illustrations of the two environments are provided in Figure 2. Table 1 provides a concise summary of the state, action, and reward definitions for both routing tasks.

In both tasks, an agent incrementally constructs a routing solution by selecting discrete actions that extend a partial route. The decision horizon grows with the problem size, and meaningful task-level feedback is only available once a complete and feasible solution has been constructed. As a result, both environments naturally induce sparse and delayed reward signals, posing a significant exploration challenge for standard reinforcement learning algorithms and making them suitable testbeds for evaluating intrinsically motivated approaches.

#### 4.1.1. Task 1: Travelling Salesman Problem

The Travelling Salesman Problem (TSP), illustrated in the left sub-figure of Figure 2, requires an agent to construct a tour that visits each city exactly once while minimizing the total travel distance. At each decision step, the agent selects the next city to visit from the set of unvisited cities, thereby extending a partial tour until a complete solution is formed.

The state observed by the agent encodes the current partial solution, including the coordinates of all cities, the index of the starting city, the current city, the number of cities visited so far, and an action mask indicating which cities remain available for selection. The action space is discrete, with each action corresponding to the index of a city to be visited next; actions associated with already visited cities are masked and cannot be selected.

The reward structure is sparse and delayed. The agent receives zero reward at all intermediate steps and only obtains a terminal reward once a valid tour has been completed. This terminal reward is defined as the negative total tour length, such that maximizing the cumulative reward is equivalent to minimizing the total travel distance.

#### 4.1.2. Task 2: Split Delivery Vehicle Routing Problem

The Split Delivery Vehicle Routing Problem (SDVRP), shown in the right sub-figure of Figure 2, generalizes the Capacitated Vehicle Routing Problem by allowing customer demands to be fulfilled over multiple visits. At each decision step, the agent selects the next customer to visit or decides to return to the depot, based on its current location and the remaining vehicle capacity. Upon visiting a customer, the delivered amount is determined by the remaining capacity, and both the customer demand and vehicle capacity are updated accordingly.

The state representation captures the current partial routing solution, including the depot location, customer locations, remaining demands for each customer, the current vehicle position, and the remaining vehicle capacity. The action space consists of discrete choices corresponding to customer indices and a special action for returning to the depot. Only feasible actions are permitted, i.e., customers whose demands can be partially or fully served given the remaining capacity, or returning to the depot when reloading is required.

As in the TSP setting, the reward signal in SDVRP is sparse and delayed. The agent receives zero reward during intermediate routing decisions and obtains a terminal reward equal to the negative total route length once all customer demands have been fully satisfied. Maximizing the cumulative reward therefore corresponds to minimizing the total travel distance while ensuring all demands are met.

To assess performance across different levels of combinatorial complexity, we evaluate each task at multiple problem scales. For the TSP, problem instances are generated with N∈{50,100,200} cities. For the SDVRP, we consider instances with {20,50,100} customers. These settings allow us to systematically examine the scalability of the proposed intrinsically motivated framework as the decision horizon and state-action space grow.

### 4.2. Baselines

We compare the proposed VIB-IG framework against a set of representative baselines commonly used in routing and combinatorial optimization. The selected baselines include standard policy-gradient methods, PPO-based variants, recent learning-based optimization approaches, as well as a classical combinatorial heuristic to provide additional performance context.

- LKH [31]: A classical Lin-Kernighan heuristic for the Traveling Salesman Problem, which represents a highly optimized, non-learning-based solver. LKH serves as a strong classical reference, helping to contextualize the performance of learning-based approaches under the same problem instances.
- PPO [36]: A widely used on-policy actor–critic algorithm that serves as our primary baseline. PPO is trained using only task-specific extrinsic rewards and provides a strong and stable reference for evaluating the benefit of intrinsic motivation.
- REINFORCE [40]: A classic Monte Carlo policy-gradient method that has been applied to routing and scheduling problems in prior work. We include REINFORCE as a minimal baseline to highlight the impact of variance reduction and actor–critic structure.
- Attention Model (AM) [10]: A state-of-the-art learning-based approach for combinatorial optimization that combines an attention-based encoder with REINFORCE. AM represents a strong task-specific baseline widely adopted in routing benchmarks.
- NeuOpt [41]: A recent reinforcement learning framework that employs a dual-aspect collaborative transformer to iteratively refine routing solutions. NeuOpt serves as a representative of modern transformer-based optimization methods.
- IBE [35]: A closely related information-theoretic reinforcement learning method that introduces both state and policy bottlenecks to regularize policy optimization. In contrast to IBE, our VIB-IG framework focuses exclusively on representation-level compression via a variational Information Bottleneck and defines intrinsic rewards through pointwise information gain, without imposing a bottleneck on the policy distribution. This design choice allows VIB-IG to decouple representation learning from policy regularization and enables a more direct investigation of information gain as an intrinsic exploration signal.

### 4.3. Implementation Details

All methods are implemented within a unified PPO-based training framework to ensure a fair comparison. Unless otherwise specified, all baselines share the same optimizer, learning rate, batch size, and training protocol.

We use the Adam optimizer with a learning rate of $10^{-4}$ and a batch size of 512. Training is performed for 10 epochs, where in each epoch trajectories are collected and gradient updates are applied until all sampled data are consumed. The discount factor and GAE parameter are set to γ=0.99 and λ=0.9, respectively. These values are kept fixed across all experiments.

For routing tasks, VIB-IG and all PPO-based baselines employ the same policy optimization objective, differing only in the reward signal and representation learning objectives. In particular, PPO and PPO-based baselines are trained using extrinsic rewards only, while VIB-IG augments the extrinsic reward with an intrinsic reward derived from information gain in the learned latent space.

#### 4.3.1. Network Architectures

For attention-based models, including VIB-IG, IBE and AM, we adopt an attention encoder with 3 transformer layers, 4 attention heads per layer, and a 128-dimensional embedding space. ReLU activations are used throughout, and no normalization layers are applied within the transformer blocks. Policy and value heads consist of fully connected layers with matching embedding dimensionality.

All non-attention-based baselines use a fully connected encoder composed of three layers with 128 hidden units each, followed by separate policy and value heads. This ensures that performance differences are attributable to algorithmic design rather than model capacity.

#### 4.3.2. Information Bottleneck Configuration

For VIB-IG, the encoder outputs the mean of a Gaussian latent distribution, with a fixed diagonal covariance. The compression strength of the variational Information Bottleneck is controlled by the coefficient β, whose effect is examined in the ablation studies (Section 4.6). The predictive information term is estimated using a neural mutual information estimator, and the resulting pointwise information gain is used to define the intrinsic reward.

Unless otherwise stated, hyperparameters for all baselines are selected to match those used in VIB-IG as closely as possible.

### 4.4. Results on the Travelling Salesman Problem

We evaluate the proposed VIB-IG framework on the Travelling Salesman Problem (TSP) across three problem scales with N=50, 100, and 200 cities. Quantitative results in terms of average tour length (lower is better) are reported in Table 2, where all values are averaged over three independent runs.

Across all problem sizes, VIB-IG consistently achieves the best solution quality among all compared methods. On TSP-50, VIB-IG attains an average tour length of 5.31±0.10, improving over the closely related IBE method (5.34±0.11) and outperforming PPO, REINFORCE, AM, and NeuOpt.

As the problem size increases to TSP-100, the advantage of VIB-IG becomes more pronounced. VIB-IG achieves an average tour length of 7.55±0.11, compared to 7.61±0.12 for IBE, while all remaining baselines converge to higher costs. This consistent improvement suggests that defining intrinsic rewards via information gain in a bottlenecked latent space yields more effective exploration than relying solely on representation regularization.

For large-scale instances with N=200, training stability becomes a critical challenge due to the long decision horizon and sparse terminal rewards. PPO and REINFORCE exhibit unstable learning dynamics with large fluctuations across runs and are therefore omitted from Table 2 for this scale. In contrast, attention-based methods such as AM and NeuOpt remain stable but converge to higher tour lengths (11.15±0.15 and 11.07±0.14, respectively). VIB-IG maintains stable learning behavior and achieves the best final performance with an average tour length of 10.82±0.12, improving over IBE (10.93±0.13).

Overall, these results indicate that information-gain-driven intrinsic rewards, when computed in an Information Bottleneck–regularized latent space, improve both solution quality and robustness as the combinatorial complexity of the routing problem increases.

### 4.5. Results on the Split Delivery Vehicle Routing Problem

We further evaluate the proposed VIB-IG framework on the Split Delivery Vehicle Routing Problem (SDVRP), a more challenging routing task characterized by vehicle capacity constraints, split deliveries, and extended decision horizons. Compared to TSP, SDVRP induces more complex state transitions and substantially sparser and more delayed reward signals. Quantitative results in terms of average total route length (lower is better) are summarized in Table 3.

On SDVRP-20, where most learning-based methods are able to learn feasible routing strategies, VIB-IG achieves the lowest average route length (5.12±0.22), improving over the closely related IBE method (5.32±0.28). Attention-based methods such as NeuOpt and AM also perform competitively but converge to higher routing costs. In contrast, PPO and REINFORCE exhibit substantially worse performance, reflecting the difficulty of exploration under sparse terminal rewards.

As the problem scale increases to SDVRP-50, performance differences become more pronounced. While attention-based methods and IBE remain relatively stable, VIB-IG consistently achieves lower routing costs (9.00±0.23) than all baselines. PPO and REINFORCE show increased variance across runs, indicating sensitivity to initialization and long-horizon credit assignment. These results suggest that intrinsic rewards based on information gain are particularly beneficial in guiding exploration when capacity constraints and split deliveries increase the complexity of the routing process.

On the largest instances (SDVRP-100), the challenges of long planning horizons and delayed rewards become even more evident. PPO and REINFORCE suffer from unstable learning dynamics and converge to substantially higher costs. Attention-based methods remain stable but learn more slowly and converge to higher route lengths. In contrast, VIB-IG maintains stable training behavior and achieves the best final performance (14.20±0.23), improving over IBE (14.60±0.37) and outperforming all remaining baselines.

Overall, the SDVRP results further corroborate the effectiveness of the proposed VIB-IG framework. By learning compact and predictive latent representations through the Information Bottleneck principle and defining intrinsic rewards via information gain, VIB-IG improves both solution quality and robustness in complex routing problems with capacity constraints and sparse, delayed rewards.

### 4.6. Ablation and Sensitivity Analysis

In this subsection, we conduct a comprehensive ablation and sensitivity analysis to better understand the role of each component in the proposed VIB-IG framework. All experiments in this section are conducted on representative problem scales, namely TSP-100 and SDVRP-50, which provide a balanced trade-off between problem complexity and computational cost. Results are averaged over three independent runs.

#### 4.6.1. Component Ablation

The proposed VIB-IG framework tightly couples representation learning and intrinsic reward computation: information gain is defined and evaluated in the bottlenecked latent space learned via the Information Bottleneck principle. As a result, standard ablation strategies that independently toggle individual components are not always conceptually meaningful. This is because the intrinsic reward is explicitly defined in the learned bottlenecked representation space, and removing either component fundamentally changes the semantics of the objective. Instead, we design ablation baselines that remove or replace components in a way that preserves the semantic interpretation of the resulting objective.

Specifically, we consider the following variants:

- PPO (no intrinsic): The vanilla PPO agent trained solely with task-specific extrinsic rewards. This baseline reflects standard policy-gradient learning under sparse terminal rewards and serves as a lower bound on performance.
- PPO + intrinsic (raw/naive latent): PPO augmented with an intrinsic reward computed either directly from raw observations or from a naively learned deterministic latent representation (implemented as a multilayer perceptron without Information Bottleneck regularization). This baseline evaluates whether intrinsic rewards alone are sufficient to improve exploration when the latent space lacks explicit structure.
- PPO + VIB (no intrinsic): PPO equipped with a variational Information Bottleneck applied to the state encoder, but without any intrinsic reward. This variant isolates the effect of representation compression and tests whether learning compact, task-relevant latent states alone can improve performance under sparse rewards.
- VIB-IG (full): The complete proposed framework, combining variational representation compression with information-gain-based intrinsic rewards computed in the learned latent space.

The quantitative results are reported in Table 4. On both TSP-100 and SDVRP-50, PPO with extrinsic rewards alone performs the worst, highlighting the difficulty of exploration under sparse terminal feedback. Adding intrinsic rewards computed from raw or naively learned representations yields modest improvements, but performance remains limited due to noisy or uninformative exploration signals.

Introducing a variational Information Bottleneck without intrinsic rewards improves training stability and solution quality, indicating that representation compression helps filter task-irrelevant variability. However, the best performance is achieved only when both components are combined. VIB-IG consistently outperforms all ablated variants, demonstrating that information-gain-based intrinsic rewards are most effective when computed in a structured, bottlenecked latent space.

#### 4.6.2. Sensitivity to the IB Coefficient β

We next analyze the sensitivity of VIB-IG to the Information Bottleneck coefficient β, which controls the trade-off between representation compression and predictive sufficiency. Intuitively, smaller values of β place less emphasis on compression and may allow task-irrelevant details to persist in the latent space, while overly large values may over-compress the representation and discard information necessary for accurate state transitions.

As shown in Table 5, setting β=0 leads to degraded performance on both tasks, confirming that explicit bottleneck regularization is beneficial. Performance improves for moderate values of β, indicating that a compact yet predictive latent representation is crucial for computing meaningful information gain. When β becomes too large, performance deteriorates, suggesting over-compression. Overall, VIB-IG exhibits stable performance over a broad range of β, indicating robustness to hyperparameter selection.

#### 4.6.3. Effect of Intrinsic Reward Weight and Scheduling

Finally, we investigate the effect of the intrinsic reward weight λ, which balances exploration driven by information gain and task optimization guided by extrinsic rewards. In addition to constant values of λ, we evaluate an annealed schedule in which λ is gradually reduced during training, allowing the agent to shift from exploration to exploitation.

The results in Table 6 show that constant intrinsic weights already improve performance over the extrinsic-only baseline. However, annealing λ consistently yields the best final solution quality. This behavior aligns with the intuition that intrinsic rewards are most useful during early exploration, while their influence should diminish as the agent converges toward high-quality solutions. These results demonstrate that intrinsic reward scheduling is an effective and practically important component of the VIB-IG framework.

Notably, annealing the intrinsic reward weight λ not only improves the mean performance but also reduces performance variance on TSP-100. This suggests that gradually shifting from exploration to exploitation leads to more consistent convergence across random seeds. On SDVRP-50, while annealing still yields the best average performance, the variance remains comparable to fixed-λ settings, likely due to the additional combinatorial flexibility introduced by split deliveries and capacity constraints.

We further conduct a sensitivity analysis on the initial intrinsic reward weight $\lambda_{0}$ under linear annealing. As shown in Table 7, varying $\lambda_{0}$ within a reasonable range (0.3–0.7) leads to comparable performance on both TSP-100 and SDVRP-50, indicating that the proposed framework is not overly sensitive to this hyperparameter. Linear annealing with the default setting achieves slightly better performance and lower variance, and is therefore adopted in all experiments.

### 4.7. Latent Space Analysis of Partial Routing Solutions

To further examine whether the proposed VIB-IG framework learns task-relevant representations beyond performance improvements, we analyze the structure of the learned latent space for partial routing solutions. This analysis directly addresses whether the Information Bottleneck–regularized latent representations capture meaningful structure associated with intermediate decision states.

In the context of routing problems, a partial routing solution refers to an intermediate state of the decision process in which only a subset of nodes has been visited and the route construction is incomplete. Such partial solutions encode rich combinatorial structure, including the set of visited nodes, the ordering of visits, and the remaining decision horizon. Understanding how these intermediate states are represented in the latent space provides insight into the agent’s internal organization of the problem.

We focus on the TSP with 50 cities (TSP-50). For each method, we collect 1000 partial routing states sampled uniformly from different training trajectories and decision stages. Each partial state is encoded into the learned latent space using the corresponding state encoder. To visualize the latent structure, we apply t-SNE to project the latent representations into two dimensions.

To assess whether structurally similar partial solutions are embedded close to each other, each latent point is colored according to the Jaccard similarity between its visited-node set and a canonical reference partial solution. This similarity measure reflects structural overlap between partial routes while remaining invariant to irrelevant ordering or geometric transformations. Higher similarity values indicate partial solutions that share a larger proportion of visited nodes with the reference solution.

**Figure 3:**
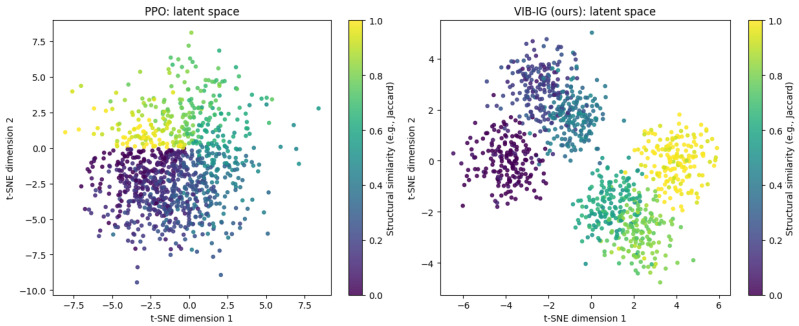
Visualization of latent representations of partial routing solutions on TSP-50 using t-SNE. Each point corresponds to a partial routing state sampled during training (1000 states per method). Points are colored by the Jaccard similarity between the visited-node set of the partial solution and a canonical reference solution with a fixed completion ratio. Compared to PPO, VIB-IG produces a more structured latent space in which structurally similar partial solutions are embedded closer together.

Figure 3 compares the latent space organization learned by PPO and by the proposed VIB-IG framework. Under PPO, latent representations exhibit weak structural organization: partial solutions with similar visited-node sets are scattered across the latent space, indicating that the learned representation does not explicitly reflect task-relevant combinatorial structure. In contrast, VIB-IG produces a more structured latent space, where partial solutions with similar structural properties are embedded closer together and form coherent clusters.

This qualitative difference suggests that the IB objective encourages the encoder to preserve task-relevant structure while suppressing nuisance variability. As a result, information gain computed in this latent space provides a more meaningful intrinsic reward signal, guiding exploration toward informative and structurally diverse partial solutions.

## 5. Conclusions and Future Work

In this work, we proposed VIB-IG, an intrinsically motivated reinforcement learning framework that integrates the Information Bottleneck principle with information-gain–driven exploration for solving combinatorial routing problems. The core idea of VIB-IG is to learn compact and predictive latent state representations via a variational Information Bottleneck, and to define intrinsic rewards directly in the learned latent space based on information gain. This design decouples representation learning from policy optimization and provides a principled, information-theoretic signal for guiding exploration under sparse and delayed rewards.

We evaluated VIB-IG on two representative routing tasks, the Travelling Salesman Problem and the Split Delivery Vehicle Routing Problem, across multiple problem scales. Experimental results demonstrate that VIB-IG consistently improves solution quality and training stability compared to standard reinforcement learning baselines, attention-based optimization methods, and closely related information-theoretic approaches. In particular, VIB-IG exhibits robust performance on larger problem instances, where long decision horizons and sparse terminal rewards pose significant challenges for exploration. Ablation studies further confirm that both variational representation compression and information-gain-based intrinsic rewards contribute meaningfully to the overall performance, and that their combination yields the most consistent improvements.

There are several promising directions for future work. In this study, the trade-off coefficients associated with the Information Bottleneck objective and the intrinsic reward are fixed throughout training. Adapting these coefficients dynamically to balance compression and exploration over time may further enhance performance. In addition, extending the proposed framework to other classes of combinatorial optimization problems and multi-vehicle routing settings represents an interesting avenue for future research.

## Figures and Tables

**Table 1:** Summary of state, action, and reward definitions for the routing tasks considered in this work.

Task	State Representation	Action Space	Reward Function
TSP	Coordinates of all cities; index of the starting city; current city; number of visited cities; action mask indicating unvisited cities.	Discrete selection of the next city to visit from the set of unvisited cities.	Sparse terminal reward equal to the negative total tour length; zero reward at intermediate steps.
SDVRP	Depot location; customer locations; remaining customer demands; current vehicle location; remaining vehicle capacity.	Discrete selection of a customer to serve or returning to the depot; only feasible actions allowed based on remaining capacity and demand.	Sparse terminal reward equal to the negative total route length once all demands are satisfied; zero reward otherwise.

**Table 2:** Performance comparison on the Travelling Salesman Problem. We report the average tour length (mean ± std over three runs; lower is better). Best results are highlighted in bold.

Method	TSP-50	TSP-100	TSP-200
LKH [31]	5.50±0.09	7.74±0.10	11.17±0.12
PPO [36]	5.56±0.24	7.88±0.27	–
REINFORCE [40]	5.47±0.21	7.82±0.25	–
AM [10]	5.49±0.13	7.74±0.14	11.15±0.15
NeuOpt [41]	5.42±0.12	7.69±0.13	11.07±0.14
IBE [35]	5.34±0.11	7.61±0.12	10.93±0.13
**VIB-IG (Ours)**	5.31±0.10	7.55±0.11	10.82±0.12

**Table 3:** Performance comparison on the Split Delivery Vehicle Routing Problem (SDVRP). We report the average total route length (mean ± std over three runs; lower is better). Best results are highlighted in bold.

Method	SDVRP-20	SDVRP-50	SDVRP-100
LKH [31]	5.49±0.20	9.72±0.20	15.42±0.23
PPO [36]	6.33±0.27	10.90±0.28	17.80±0.30
REINFORCE [40]	6.51±0.32	11.40±0.35	18.50±0.38
AM [10]	5.49±0.21	9.70±0.21	15.40±0.25
NeuOpt [41]	5.42±0.23	9.50±0.24	15.10±0.25
IBE [35]	5.32±0.28	9.20±0.33	14.60±0.37
**VIB-IG (Ours)**	5.12±0.22	9.00±0.23	14.20±0.23

**Table 4:** Component ablation results on TSP-100 and SDVRP-50 (mean ± std over three runs; lower is better).

Method	TSP-100	SDVRP-50
PPO (no intrinsic)	7.88±0.27	10.90±0.28
PPO + intrinsic (raw/naive latent)	7.74±0.24	10.30±0.22
PPO + VIB (no intrinsic)	7.63±0.12	9.60±0.20
VIB-IG (full)	7.55±0.11	9.00±0.23

**Table 5:** Sensitivity to the Information Bottleneck coefficient β on TSP-100 and SDVRP-50 (mean ± std over three runs). In our implementation, the default choice is β=0.01, highlighted in bold.

Task	β=0	$10^{-3}$	$10^{-2}$	$10^{-1}$	1
TSP-100	7.78±0.14	7.66±0.10	7.55±0.11	7.62±0.13	7.81±0.15
SDVRP-50	9.85±0.25	9.42±0.22	9.00±0.23	9.15±0.20	9.55±0.24

**Table 6:** Effect of intrinsic reward weight λ and scheduling on TSP-100 and SDVRP-50 (mean ± std over three runs).

Setting	TSP-100	SDVRP-50
λ=0	7.92±0.28	10.90±0.25
λ=0.3	7.68±0.22	9.55±0.22
λ=0.5	7.60±0.20	9.25±0.20
Annealed λ (ours)	7.55±0.11	9.00±0.23

**Table 7:** Sensitivity analysis of the initial intrinsic reward weight $\lambda_{0}$ under linear annealing (mean ± std over three runs).

Setting (Linear Annealing)	TSP-100	SDVRP-50
$\lambda_{0}=0.3\to 0$	7.58±0.13	9.10±0.24
$\lambda_{0}=0.5\to 0$ (default)	7.55±0.11	9.00±0.23
$\lambda_{0}=0.7\to 0$	7.57±0.11	9.05±0.26

## Data Availability

The data used in this study were generated using publicly available benchmark environments for combinatorial optimization, including the Travelling Salesman Problem (TSP) and the Split Delivery Vehicle Routing Problem (SDVRP). All datasets are synthetic and can be fully reproduced by following the experimental setup and parameter configurations described in the paper. The code used to generate the data and conduct the experiments is available from the corresponding authors upon reasonable request.
